# Epidemiology of Wilson disease in Germany – real-world insights from a claims data study

**DOI:** 10.1186/s13023-024-03351-2

**Published:** 2024-09-11

**Authors:** Shona Fang, Peter Hedera, Julia Borchert, Michael Schultze, Karl Heinz Weiss

**Affiliations:** 1 Alexion, AstraZeneca Rare Disease, 121 Seaport Avenue, Boston, 02210 MA USA; 2 University of Louisville, Louisville, KY USA; 3 Scientific Institute for Health Economics and Health System Research (WIG2 GmbH), Leipzig, Germany; 4 Berlin Center for Epidemiology and Health Research, ZEG Berlin GmbH, Berlin, Germany; 5 Salem Medical Center, Heidelberg, Germany

**Keywords:** Wilson disease, Epidemiology, Prevalence, Real-world evidence, Claims data

## Abstract

**Background:**

Wilson disease (WD) is a rare disorder of copper metabolism, causing copper accumulation mainly in the liver and the brain. The prevalence of WD was previously estimated around 20 to 33.3 patients per million for the United States, Europe, and Asia, but data on the prevalence of WD in Germany are limited.

**Objectives:**

To describe patient characteristics and to assess prevalence of WD in Germany using a representative claims database.

**Methods:**

WD patients were identified in the WIG2 (*Wissenschaftliches Institut für Gesundheitsökonomie und Gesundheitssystemforschung;* Scientific Institute for Health Economics and Health Systems Research) benchmark database of 4.5 million insured Germans by combining ICD-10-coding with WD-specific lab tests and treatments. The study period ranged from 2013 to 2016 for assessing patient characteristics, and to 2018 for prevalence, respectively.

**Results:**

Seventy unique patients were identified. Most patients (86%) were between 18 and 64 years of age and more often male (60%) than female. Two patients (3%) younger than 18 years were included, as well as 8 patients (11%) older than 64 years. Most common WD subtypes were hepatic (57%), psychiatric (49%), and neurologic (44%). Average prevalence was 20.3 patients per million (range: 17.8–24.4), with similar results for two-year prevalence. Generally, prevalence increased steadily over the study period. Observed mortality was low, with only one death during the study period.

**Conclusions:**

This study adds valuable real-world data on the prevalence and patient characteristics of WD in Germany. Generally, our findings align with other reports and contribute to the global understanding of WD epidemiology. Still, regional and temporal trends remain to be investigated more thoroughly to further the understanding of the natural history and epidemiology of this rare disease.

## Background

Wilson disease (WD) is a rare autosomal recessive disorder of copper metabolism [1], caused by biallelic mutations in homo- or compound heterozygous states in the *ATP7B* gene [2, 3]. While copper is usually metabolized by hepatocytes or excreted via bile into feces when present in excess, copper will accumulate in WD patients, mostly in the liver and the brain, although other organs may be affected as well [4, 5]. Depending on the severity and the organs involved, the build-up of copper can lead to a rather broad variety of manifestations, such as liver disease, renal tubular acidosis, or movement or cognitive disorders. Deposition of copper in the Desçemet’s membrane may lead to Kayser-Fleischer rings, which are present in about 50% of WD patients with liver disease and almost all patients with neurologic or psychiatric manifestation of WD [5]. A diagnosis of WD may be made with the combined presence of Kayser–Fleischer rings and low serum ceruloplasmin level (< 0.1 g/L) [6]. The disease is currently not curable, but it is well manageable with a copper-restrictive diet and pharmacological treatment to minimize the build-up of copper. Usually, treatment consists of chelators (such as D-penicillamine or trientine) to promote the renal excretion of copper, and zinc to block intestinal copper absorption [5, 7]. Due to the variation in the organs affected and the accompanying symptoms, symptomatic WD can be categorized into three main overlapping subtypes according to the cardinal symptoms: hepatic, neurologic, and/or psychiatric. Neurologic manifestations in particular can lead to severe disability as patients may experience increasing difficulty in controlling movement or progressive dystonia [6]. Despite the availability of treatment options, subpar adherence to the treatment has been reported especially in pediatric and asymptomatic patients [8]. Considering the potentially fatal risks of insufficient treatment, disease awareness to increase adherence is critical [8, 9].

WD is considered a rare disease: besides regional exceptions [10], the prevalence of WD is estimated around 20 to 33.3 patients per million in the United States, Europe and Asia [11], although prevalence data on national levels vary with regard to region and time period [12–15]. Also, considering the broad range of subtypes and associated symptoms, more detailed assessments regarding epidemiology are needed for a better understanding of the patient population and to improve disease management. For Germany, recent publications have addressed the treatment of WD [16] and estimated prevalence to be around 16.7 patients per million [17]. We aim to complement and update these findings with more comprehensive data from an observational and representative claims-based study by assessing patient characteristics and epidemiology of WD in Germany.

## Methods

### Study design

The study was a retrospective, observational study using a claims database to assess patient characteristics and prevalence of WD from a national perspective in Germany. The overall study period included data from the beginning of 2011 to the end of 2018. Study cohorts were identified between the start of 2013 and the end of 2016 (index period), resulting in a pre- and post-index period of two years each, with an observational period from 2013 to 2016 for describing population characteristics and from 2013 to 2018 for assessing crude annual and two-year prevalence.

### Population

The study population consisted of patients diagnosed with WD during the index period. To assess prevalence, patients had at least one diagnosis in outpatient or inpatient visits during the respective year and at least one main inpatient or two outpatient (or secondary inpatient) diagnoses during the study period (2011–2018), with a minimum of two years pre-index required. For infants younger than two years of age, the pre-index period may be less than two years and in these cases, the pre-index period started from birth. All available post-index years were considered with no restrictions regarding the length of the post-index period.

The ICD-10-GM code (International Classification of Diseases, 10th version, German modification) used to identify WD patients (E83.0) does not specifically refer to WD and includes other disorders of copper metabolism (e.g., Menkes disease). Therefore, in addition to the ICD-10-GM code, all patients in question were to receive either WD medication or at least one laboratory test of both copper and ceruloplasmin, at any time point available. Any prescription WD medication was considered, including D-penicillamine, trientine, and zinc. Treatments with zinc products were included if they used lower dosages than recommended in the guidelines [6] or when using over-the-counter (OTC) products, provided zinc had been prescribed by a physician. Laboratory tests were identified with EBM codes (*Einheitlicher Bewertungsmaßstab*, uniform assessment standard for outpatient care in Germany. 32277: copper; 32440: ceruloplasmin), both of which had to be detectable within 30 days at least once per patient during the study period. No exclusion criteria, (e.g., age or gender) were applied.

### Subtype definitions

Patients were assigned to the most common WD subtypes according to comorbidities reported in the database observed at any time during the study period. Patients of the hepatic subtype were required to have a diagnosis for liver signs and symptoms, such as acute hepatitis (not viral), cirrhosis (decompensated or compensated), liver failure, portal hypertension, or hepatocellular carcinoma. Similarly, patients of the neurological subtype had to have diagnoses for tremor, Parkinsonism or akinetic rigid syndrome, gait abnormalities/ataxia, dysarthria, dystonia, chorea, dysphagia, myopathy, seizures, migraine, somatoform autonomic dysfunction, or cognitive disorders. The psychiatric subtype was assigned to patients with diagnoses for mood disorders, paranoia/schizophrenia, psychosis, or personality disorders. WD subtypes were not mutually exclusive; accordingly, patients could be assigned to one or more subtypes. Finally, the most common manifestations within subtypes were assessed.

### Outcomes

The primary objective of this study was to assess the overall, age-, and sex-specific annual prevalence of WD in Germany. A two-year prevalence was used as sensitivity analysis. Demographic patient characteristics at baseline including age, gender, their physician’s specialty practice area, treated or untreated, and distribution of WD subtype and manifestations were also collected.

### Data sources

The study used the benchmark database from the Scientific Institute for Health Economics and Health Systems Research (Wissenschaftliches Institut für Gesundheitsökonomie und Gesundheitssystemforschung [WIG2]), which comprises longitudinal data on 4.5 million insured German citizens with full billing information for utilized health services in hospitals, the ambulatory sector, and pharmaceuticals, and is considered representative of the German population insured via statutory health insurance (SHI) [18]. Claims data are made available to research by insurances with a one-year lag; data through 2018 were the most current data available at the time of this study.

### Data analysis

All statistical analyses were descriptive and exploratory and were conducted for baseline and outcome measures in the study cohorts and sub-groups. For continuous variables, the number of subjects (N), mean (SD, minimum and maximum, and the 95% confidence intervals (CI) are presented. For categorical variables, the number of subjects N and proportion in each category are presented. All outcomes are reported by age and gender at the population level. Regional differences were not detectable in a meaningful way due to the small sample size. All analyses were implemented using Microsoft structured query language (SQL) Server 2016 and R (Version 4.0.3).

## Results

### Baseline characteristics, overall and by subtype

Twenty (20) of 70 identified patients had their first WD diagnosis during the study period. The mean baseline age was 43.4 years (SD = 17.3 years), with most patients in the groups of 40 to 64 years of age (*n* = 35; 50%) and 18 to 39 years of age (*n* = 25; 36%). Considerably fewer children and adolescents (*n* = 2; 3%), as well as patients older than 65 years (*n* = 8; 11%) were observed. The mean follow-up duration among all patients was 5.11 years (SD = 1.3 years).

The majority of patients were male (*n* = 42, 60%) and most patients (*n* = 50; 71%) had received their initial WD diagnosis by a general practitioner. Of the 70 patients, 17 (24%) did not have a claim for a WD specific treatment according to the records. These 17 patients had a mean follow-up time of 5.05 years (SD = 1.15 years). The predominant subtype across all patients was the hepatic subtype comprising 40 patients (57%). Neurologic and psychiatric subtypes were detected in 31 (44%) and 34 (49%) patients, respectively. The patient characteristics at baseline and most common subtypes are given in Table 1. Across all subtypes, liver signs and symptoms were the most frequent manifestation of WD, occurring in 38 patients (54%), followed by mood disorders (*n* = 29, 41%), and osteoarthritis (*n* = 22, 31%). A list of the most common manifestations by subtype is provided in Table 2.

### Prevalence overall

Between the years 2013 and 2018, the crude annual prevalence was determined on an average sample size of 64 patients (range: 57 to 74 patients), equating to an average prevalence of 20.3 patients per million in Germany (range: 17.8 to 24.4 per million). A similar picture emerged in assessing two-year prevalence in the same study period, identifying on average 73 patients (range: 68 to 81 patients), corresponding to an average of 21.9 patients per million (range: 20 to 25.2 per million). An overview of annual prevalence is given in Table 3.

### Prevalence by gender and age

In the observed prevalent patients, men appeared more frequently affected by WD than women, with an average prevalence of 23 patients per million compared with a mean prevalence of 17 patients per million (24 and 20 per million; respectively, for two-year prevalence). The proportion of male patients in the prevalent population varied between 55.4% and 63.5% during the observed study period.

When accounting for age distribution, the prevalence estimates varied between observed age groups. The highest prevalence was seen in the age group 18 to 39 years with a mean prevalence across all study years of 26 patients per million, ranging from 17.9 to 37.2 per million. Similarly, prevalence was high in the age group 40 to 64 years with a mean of 25 patients per million across study years (range: 22.6 to 27.4 per million). In the other age groups, WD patients were less frequently observed. Among children and adolescents (0 to 17 years), the observed mean prevalence decreased to 12.6 patients per million (range: 8.5 to 17.2 per million). Mean prevalence was higher in patients aged 65 years and older (8.6 patients per million), steadily increasing and ranging from 3.6 to 12.8 per million over the study period. Similar patterns of age-related prevalence could be observed when assessing two-year prevalence.

Age-adjusted annual prevalence ranged between 17 and 24 patients per million between 2013 and 2018 (age adjusted to the German population). The two-year prevalence in the same period ranged between 19.1 and 24.6 patients per million. In both the annual and two-year prevalence, the lowest values were detected at the beginning of the study period, whereas the highest values were detected at the end of the study period in 2018.

Similarly, age-adjusted annual prevalence ranged between 17.3 and 24.6 patients per million, and between 19.8 and 25.6 patients per million for the two-year prevalence, when age adjusting to the World Health Organization (WHO) standard. Again, lowest values were detected at the beginning of the study period in 2013, highest values were detected at the end of the study period in 2018.

### Mortality

Mortality among WD patients was low, with only one death in 2014 reported during the study period. Even when considering a broader inclusion definition, by only using the corresponding ICD-10-GM code for patient identification alone, 8 deaths were reported during the study period. However, this patient population ranging from 133 to 144 prevalent patients between 2013 and 2018 may have included non-WD patients, as the underlying ICD-10-GM code was not specific to WD.

## Discussion

### Patient characteristics

Of 70 individual patients observed, 42 (60%) were male. While this slight predominance of WD among men generally aligns with previous observations [19, 20], no reasonable conclusions might be drawn from this, as gender-related aspects of WD still appear understudied and the study population observed here was quite small.

Regarding age, most patients were observed in the adult age groups between 18 and 64 years, whereas only two patients (3%) were observed among children and adolescents. Researchers had previously discussed [21, 22], that WD in children and adolescents may be underdiagnosed due to limited disease awareness among both, patients and their caregivers, and difficulties in the diagnosis. This is especially evident when adolescent patients present with neuropsychiatric symptoms that may often not be directly attributed to WD [23], as opposed to more apparent (but still not obligatory) symptoms such as Kayser-Fleischer rings [24]. On the other hand, only a few patients (*n* = 8; 11%) were observed in the age group from 65 years and older, arguably due to limited awareness in the elderly patient and due to a higher mortality in this age group in undiagnosed patients. While WD is generally treatable when diagnosed early [25], survival of WD patients is lower than in the general population as demonstrated in a long-term observational study in Austria [26]. Still, in the prevalent cohort presented here, observed mortality was very low, and annual prevalence in the oldest age group even appeared to increase over time. While this observation could suggest increased survival among older patients, considering the low number of patients it must remain speculative, nevertheless.

### Subtypes

More than half of the observed patient population (57%) presented with hepatic manifestations, which is not unexpected considering the role of the liver in copper metabolism [4] and the observation that liver symptoms commonly precede neurologic manifestations [27]. As patients often presented with multiple manifestations, many patients were also observed in the psychiatric (49%) and neurologic (44%) subtype, to which diagnostic delay has previously been ascribed [28, 29].

### Prevalence

We observed a mean prevalence of 20.3 patients per million, which steadily increased from 17.8 to 24.4 per million during the study period from 2013 to 2018, and was also observed when assessing two-year prevalences. While possibly influenced by general variance, an actual increase in documented prevalence may be conceivable due to improved awareness of rare diseases and improvements in diagnostic methods and treatment options [30, 31]. Additional studies are needed to determine whether a trend of increasing prevalence continues into the current day, however similar increases in prevalence have been described in other countries [10].

While we observed slightly higher prevalence than previously estimated for Germany [17], our findings align with other reports of WD epidemiology at large. Poujois and colleagues reported a crude annual prevalence of 15 patients per million in France [20]. Similar to the results presented here, their findings were based on insurance claims data, albeit from a larger database that covered approximately 86% of the French population. Despite this, the lower prevalence reported there is based on a shorter identification period between 2011 and 2013 and may also be subject to annual variation. For the UK, a 2021 publication reported an average prevalence of 15.5 per million between 2011 and 2018 and used a similar method to the one presented here, by combining the ICD10-code E83.0 with further lab tests from claims data to ascertain identification of WD patients [32]. With 4.5 patients per million, Sipilä and colleagues reported a distinctly smaller WD prevalence for Finland, who also discussed potential underdiagnosis in the light of genetic analyses suggesting much higher prevalence [33]. Markedly higher prevalence on the other hand, has been reported from South Korea with an average of 38.7 patients per million between 2010 and 2016, with a steady increase over time from 28 to 48.1 patients per million, arguably due to improved detection and higher survival rates [34]. A similar pattern with increasing prevalences over time was also reported from Hong Kong, albeit with slightly lower overall prevalence: Cheung and colleagues reported an average prevalence of 17.9 patients per million between 2000 and 2016, again with a steady increase from 7.8 to 25.2 per million [35].

Differences in methodologies hampers direct comparisons and may also obscure any distinction of actual differences. Also, as genetic analyses suggest [36], the global prevalence of WD might be even higher than observed in epidemiology-based reports, indicating that WD may still be severely underdiagnosed.

### Limitations

The claims data used in this study was originally recorded for reimbursement and was not collected with research purposes in mind. Therefore, it has inherent limitations, such as possible coding misclassifications, which are ultimately at the discretion of the treating physician. Also, clinical details are not included in the data, such as, e.g., cause of death or potential linkages between renal or liver failure and WD medication, or any other side effects of medications in general. Furthermore, patients were detected in the dataset during in- or outpatient visits, via prescriptions, and lab tests, potentially leading to an underestimation as asymptomatic patients may not be detected at all []. Despite the large database and inherent with a rare disease, any trends detected in this small population must be interpreted with caution. Still, it seems that our findings presented here are more likely to under- than overestimate the occurrence of WD in Germany due to possibly undetected, asymptomatic patients and in the light of genetic analyses, which suggest a higher prevalence [36]. For instance, pediatric patients may be underrepresented in our study, as the age at study entry may not necessarily have been the age at diagnosis. Despite the size of the claims database, detecting a rare disease throughout age groups may require even larger datasets, which might be further amplified by the relatively short follow-up duration. Further, we were not able to observe claims for OTC zinc products that had not been prescribed by a physician, which may account for a portion of the 24% of patients with no observed claim for a WD specific treatment. Finally, regional exceptions of increased WD prevalence are known to occur for isolated populations due to consanguinity [10]. We were unable however to study sub-populations or regions within Germany, or ascertain any impact of consanguinity on our prevalence estimate within Germany.

### Strengths

The findings presented here are based on a relatively large and nationally representative database. In addition to a longitudinal view of patients, the main advantage of the WIG2 database is the completeness of available data, as both in- and outpatient sectors, as well as prescribed drugs (including OTC zinc if prescribed by a physician) are available in their entirety, provided they are invoiced via the SHI system. Our diagnostic algorithms (based on diagnostic code, prescriptions, and treatments) may improve sensitivity. Thus, the study provides a rather accurate account of WD in Germany and contributes to a slowly growing body of evidence in WD epidemiology, improving our understanding of this rare disease. Despite a potential underestimation, the patient characteristics and prevalence reported here are based on real-world data, and thus provide insights into clinical reality.

**Table 1:** Demographic characteristics at baseline and by subtype for prevalent cohort

	No subtype (*n* = 3)	Liver manifestations (*n* = 40)	Neurologic manifestations (*n* = 31)	Psychiatric manifestations (*n* = 34)	Overall (*n* = 70)
**Age at index date (years)**					
Mean (SD)	26.7 (4.0)	41.3 (17.9)	46.4 (17.0)	44.9 (17.9)	43.4 (17.3)
**Age at index date (categorical); n (%)**					
0–17	0 (0%)	1 (3%)	1 (3%)	1 (3%)	2 (3%)
18–39	3 (100%)	16 (40%)	9 (29%)	13 (38%)	25 (36%)
40–64	0 (0%)	19 (48%)	18 (58%)	15 (44%)	35 (50%)
≥ 65	0 (0%)	4 (10%)	3 (10%)	5 (15%)	8 (11%)
**Gender; n (%)**					
Female	1 (33%)	17 (43%)	11 (35%)	12 (35%)	28 (40%)
Male	2 (67%)	23 (58%)	20 (65%)	22 (65%)	42 (60%)
**Follow-up period (years)**					
Mean (SD)	6.0 (–)	4.8 (1.4)	5.3 (1.2)	5.2 (1.3)	5.1 (1.3)
**Calendar year of index date; n (%)**					
2013	3 (100%)	28 (70%)	24 (77%)	23 (68%)	52 (74%)
2014	0 (0%)	3 (8%)	4 (13%)	6 (18%)	7 (10%)
2015	0 (0%)	7 (18%)	3 (10%)	4 (12%)	9 (13%)
2016	0 (0%)	2 (5%)	0 (0%)	1 (3%)	2 (3%)
Physician specialty seen at index*; n (%)					
General Practitioner	2 (67%)	26 (65%)	21 (68%)	24 (71%)	50 (71%)
Other	0 (0%)	13 (33%)	11 (35%)	11 (32%)	18 (26%)
Gastroenterologist	0 (0%)	3 (8%)	2 (6%)	2 (6%)	5 (7%)
Neurologist	0 (0%)	2 (5%)	5 (16%)	2 (6%)	5 (7%)
Pediatrician	1 (33%)	3 (8%)	1 (3%)	2 (6%)	5 (7%)
Unknown	0 (0%)	4 (10%)	1 (3%)	1 (3%)	4 (6%)
Psychologist	0 (0%)	1 (3%)	1 (3%)	1 (3%)	1 (1%)
Inpatient	0 (0%)	7 (18%)	2 (6%)	3 (9%)	7 (10%)
**No claim for WD-specific treatment; n (%)**	0 (0%)	11 (28%)	9 (29%)	9 (26%)	17 (24%)

IQR: interquartile range; SD: standard deviation

*Not necessarily the physician of WD diagnosis

**Table 2:** **A–D**: prevalent cohort – up to 20 most frequent manifestations by subtype

A: Liver manifestations (*n* = 40)			B: Neurologic manifestations (*n* = 31)			C: Psychiatric manifestations (*n* = 34)			D: Manifestations overall (*n* = 70)	
Subtype comorbidities, n (%)			Subtype comorbidities, n (%)			Subtype comorbidities, n (%)			Subtype comorbidities, n (%)	
Liver signs and symptoms	38 (95%)		Ataxia and gait abnormalities	12 (39%)		Mood disorders	29 (85%)		Liver signs and symptoms	38 (54%)
Cirrhosis	19 (48%)		Cognitive deficits	9 (29%)		Personality disorders	6 (18%)		Mood disorders	29 (41%)
Portal hypertension	11 (28%)		Tremor	9 (29%)		Mix of symptoms, children	5 (15%)		Bone, osteoarthritis	22 (31%)
Hepatitis	10 (25%)		Autonomic disfunction	6 (19%)		Paranoia and schizophrenia	4 (12%)		Cirrhosis	19 (27%)
Liver failure, undefined	9 (23%)		Dysarthria	5 (16%)		Psychosis	3 (9%)		Ataxia and gait abnormalities	12 (17%)
Liver failure, acute	2 (5%)		Dysphagia	5 (16%)					Blood, coagulopathy	11 (16%)
Liver cancer	1 (3%)		Migraine	5 (16%)					Portal hypertension	11 (16%)
			Myopathy	4 (13%)					Hepatitis	10 (14%)
			Seizures	4 (13%)					Cardiac, arrhythmia	10 (14%)
			Bradykinesia and rigidity	4 (13%)					Cognitive deficits	9 (13%)
			Dystonia	2 (6%)					Liver failure, undefined	9 (13%)
			Chorea	1 (3%)					Renal failure	9 (13%)
									Tremor	9 (13%)
									Blood, thrombocytopenia	8 (11%)
									Skin, lipomas	7 (10%)
									Autonomic disfunction	6 (9%)
									Personality disorders	6 (9%)
									Bone, demineralization	5 (7%)
									Dysphagia	5 (7%)
									Dysarthria	5 (7%)

**Table 3:** Annual prevalence of WD in Germany between 2013 and 2018

Year	2013		2014		2015		2016		2017		2018	
	*N*	Prevalence per million (95% CI)	*N*	Prevalence per million (95% CI)	*N*	Prevalence per million (95% CI)	*N*	Prevalence per million (95% CI)	*N*	Prevalence per million (95% CI)	*N*	Prevalence per million (95% CI)
**Age group (years)**												
0 to 17	8	15.3 (6.6–30.1)	9	17.2 (7.9–32.7)	6	11.7 (4.3–25.5)	6	12 (4.4–26.1)	4	8.5 (2.3–21.8)	5	11 (3.6–25.6)
18 to 39	16	17.9 (10.3–29.1)	17	19 (11.1–30.4)	24	27.3 (17.5–40.6)	21	24.5 (15.2–37.5)	25	31.2 (20.2–46)	29	37.2 (24.9–53.4)
40 to 64	31	25.2 (17.2–35.8)	32	25.8 (17.6–36.4)	28	22.6 (15–32.7)	28	22.8 (15.1–32.9)	31	26.4 (18–37.5)	32	27.4 (18.7–38.7)
≥65	2	3.6 (0.4–13.1)	4	7 (1.9–17.9)	5	8.5 (2.7–19.8)	5	8.2 (2.7–19.2)	7	11.5 (4.6–23.6)	8	12.8 (5.5–25.3)
**Gender**												
Female	24	15.8 (10.1–23.5)	25	16.2 (10.5–24)	23	15 (9.5–22.5)	25	16.4 (10.6–24.2)	27	18.5 (12.2–26.9)	33	22.8 (15.7–32)
Male	33	19.7 (13.6–27.7)	37	21.9 (15.4–30.1)	40	23.7 (16.9–32.3)	35	21 (14.6–29.2)	40	25.1 (17.9–34.1)	41	25.9 (18.6–35.2)
**Overall**	**57**	**17.8 (13.5–23.1)**	**62**	**19.2 (14.7–24.6)**	**63**	**19.5 (15–25)**	**60**	**18.8 (14.3–24.2)**	**67**	**21.9 (17–27.9)**	**74**	**24.4 (19.2–30.7)**

CI: confidence interval; WD: Wilson disease

### Algorithm for patient identification

The unambiguous identification WD patients was not possible via ATC or ICD-10-GM codes alone. Prescriptions of copper replacements were not detectable via ATC codes, as they are produced individually and are administered parenterally in combination with electrolytes as standard therapy (copper histidine). Therefore, a distinction between Menkes disease, copper deficiency, and WD based on copper replacement prescriptions alone was not possible. Nevertheless, Menkes disease and WD usually manifest at different ages: while incidence and life expectancy for Menkes disease are low, symptoms and thus, the diagnosis of WD usually occur later in life. Furthermore, as the corresponding ICD-10-GM code (E83.0) also includes other disorders of copper metabolism, an algorithm combining the ICD-10-GM code with evidence for WD specific laboratory tests or treatments was developed to detect WD patients specifically. Although this approach is not validated, our findings of annual and two-year prevalence are in line with other reports [11], and suggest that the patient identification is more accurate than solely using the ICD-10-GM code. The additional reassurance of the diagnosis via performed lab tests may further decrease a potential underestimation of WD prevalence due to asymptomatic cases [36].

## Conclusions

In summary, this study adds valuable real-world data on the prevalence and patient characteristics of WD in Germany. Generally, our findings align with other reports and contribute to the global understanding of WD epidemiology. Still, regional and temporal trends remain to be investigated more thoroughly to further the understanding of the natural history and epidemiology of this rare disease.

## Data Availability

The datasets generated and analyzed in this study are not publicly available due to data protection laws. Raw data are not publicly available to preserve individuals’ privacy under the European General Data Protection Regulation.
